# Dendritic Macrosurfactant Assembly for Physical Functionalization of HIPE-Templated Polymers

**DOI:** 10.3390/polym12040779

**Published:** 2020-04-01

**Authors:** Chenhui Li, Shiqi Weng, Ming Jin, Decheng Wan

**Affiliations:** Department of Polymer Materials, School of Materials Science and Engineering, Tongji University, 4800 Cao-an Rd, Shanghai 201804, China; 1710814@tongji.edu.cn (C.L.); wsq8030@163.com (S.W.); mingjin@tongji.edu.cn (M.J.)

**Keywords:** polyethyleneimine, dendritic macrosurfactant, porous organic polymer, functional surface, adsorption

## Abstract

High-internal-phase emulsion-templated macroporous polymers (polyHIPEs) have attracted much interest, but their surface functionalization remains a primary concern. Thus, competitive surface functionalization via physical self-assembly of macrosurfactants was reviewed. Dendritic and diblock-copolymer macrosurfactants were tested, and the former appeared to be more topologically competitive in terms of solubility, viscosity, and versatility. In particular, hyperbranched polyethyleneimine (PEI) was transformed into dendritic PEI macrosurfactants through click-like *N*-alkylation with epoxy compounds. Free-standing PEI macrosurfactants were used as molecular nanocapsules for charge-selective guest encapsulation and robustly dictated the surface of a macroporous polymer through the HIPE technique, in which the macroporous polymer could act as a well-recoverable adsorbent. Metal nanoparticle-loaded PEI macrosurfactants could similarly lead to polyHIPE, whose surface was dictated by its catalytic component. Unlike conventional Pickering stabilizer, PEI macrosurfactant-based metal nanocomposite resulted in open-cellular polyHIPE, rendering the catalytic sites well accessible. The active amino groups on the polyHIPE could also be transformed into functional groups of aminopolycarboxylic acids, which could efficiently eliminate trace and heavy metal species in water.

## 1. Introduction

Given the potential of high-internal-phase emulsion (HIPE)-templated macroporous polymers (polyHIPEs) in adsorption [1,2,3,4], catalysis [5,6,7,8], bioapplication [9], and separation [10] due to its invention [11], these polymers have been well reviewed by a number of researchers [12,13,14,15,16,17]. HIPE is defined as an emulsion whose internal phase makes up over 74% of the total system volume. HIPE can be prepared by either dispersing water in oil (W/O), oil in water (O/W), air in oil, or supercritical carbon dioxide in oil. Among these techniques, W/O emulsion is the most widely studied, partly because of easy access to green water. In the present study, HIPE was prepared via W/O unless stated otherwise. PolyHIPEs are widely explored due to their cost-effective preparation, relatively large specific surface area, interconnected pores, and wide range of potential applications. These applications are largely determined by their surface functions. Surface functionalization of polyHIPEs can be performed using intrinsic functional monomers, through chemical postmodification of the matrix, or using functional surfactants, where the last method is less explored. Small surfactants are not recommended for surface functionalization, because they can be readily washed away, but reactive surfactants cannot [18,19]. Surface functionalization of polyHIPE can be performed via self-assembly of macrosurfactants, especially of dendritic macrosurfactants, which are less studied and have not been reviewed. Emulsion type is also critical to the surface properties of polyHIPEs. For example, a W/O HIPE should be adopted to achieve a polyHIPE with a hydrophilic surface and if the hydrophilic block rather than the hydrophobic block of an amphiphilic block copolymer must dictate the surface of the polyHIPE. The Bancroft rule, which states that “the phase in which the surfactant is most soluble constitutes the continuous phase,” [20] is valid in most cases. Thus, an organo-soluble macrosurfactant should be used to obtain W/O emulsions. This review focuses on the surface functionalization of polyHIPEs via self-assembly of acid- and base-durable dendritic macrosurfactants for polyHIPE recycling.

Different from linear, grafted, and crosslinking polymers, dendritic polymers consist of dendrimers and hyperbranched polymers with topology-related properties. They exhibit globular topology, rich terminal groups, small sizes, and rare or no chain entanglement. They also have lower viscosity and better solubility than their linear counterparts in conventional solvents. Either dendrimer derivatives [21] or hyperbranched macrosurfactants (also called branched polymersomes) [22] can form nanostructures via self-assembly. This technique has been extensively studied for biomedical applications, chemical nanoreactors, and host–guest chemistry [23,24]. Polyethyleneimine (PEI) is one of several commercialized hyperbranched polymers well known for their dense active amino groups. It is a hydrophilic polyamine polymer, which can be transformed into a reverse micelle-like PEI macrosurfactant by alkylation with long hydrophobic chains. PEI macrosurfactants are also called molecular nanocapsules due to their covalent nature [25,26,27,28,29,30,31,32,33,34,35,36,37]. These nanocapsules can undergo guest encapsulation via liquid–liquid phase-transfer extraction [26,27,28,29,30,31,32,33,34,35,36,37]. Unlike dendrimers, PEI macrosurfactants rarely show topological selectivity over guest sizes [33] but widely show charge-selective encapsulation [32,33,34,35]. Fine guest selectivity [36] and controlled guest release [37] have been observed in molecular nanocapsules through core engineering.

PEI macrosurfactants are used to dictate the surface of HIPE-templated polymers. Unlike a free-standing molecular nanocapsule in solvent, a supported macrosurfactant can easily be recovered, thereby not causing secondary pollution during wastewater treatment. Polymerizing the continuous phase of HIPE is a classic method used to prepare macroporous polymers [12,13,14,15,16,17]. However, the surface of a typical polyHIPE is typically dictated by small surfactants, which are readily eroded away. A more permanent monolayer functional surface can be obtained when an amphiphilic block copolymer is used in place of a small surfactant [38,39,40,41]. The drawbacks of diblock copolymer macrosurfactants include high viscosity and/or a strong tendency to aggregate in the monomer phase of a HIPE. Star copolymers [42] or heterografted copolymers [43] are highly efficient emulsion stabilizers. The dendrimers of polypropyleneimine or polyamidoamine have been used along with a small surfactant to mediate the emulsion polymerization system [44,45]. Dendritic macrosurfactant applications in the HIPE system have been reported only recently [3]. Dendritic macrosurfactants typically show much lower viscosity and better solubility than their linear counterparts [2]. The synthesis and host–guest properties of PEI macrosurfactants are explored in detail in the subsequent sections. Applying PEI macrosurfactant to polyHIPE could combine the functional versatility of PEI macrosurfactant with the large-scale production of porous polyHIPE, thereby extending the ordered surface to macroscopic scale.

## 2. Synthesis of PEI Macrosurfactants

**Figure 1:**
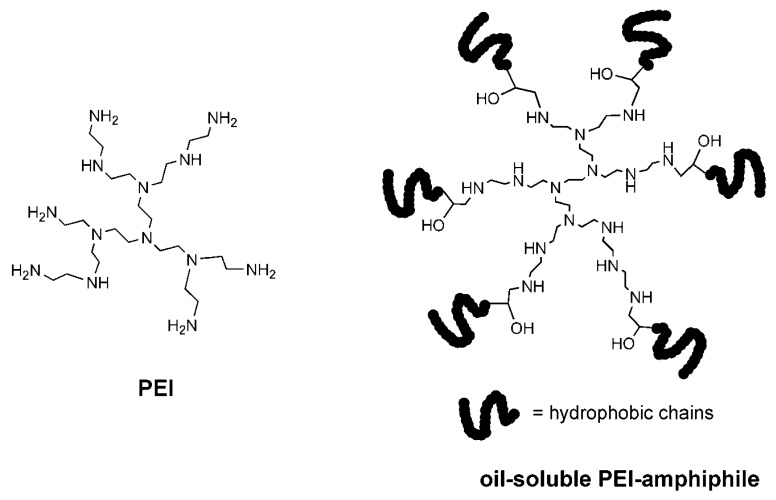
Chemical structures of polyethyleneimine (PEI) and typical oil-soluble PEI macrosurfactant. PEI contains thousands of repeat units, but only some are shown here for clarity.

PEI contains dense primary, secondary, and tertiary amino groups (Figure 1). Amino groups from small molecules are reactive and readily modified by chemical reactions, but PEIs are not. Upon encountering reactants such as fatty acid chlorides or fatty acid anhydrides, PEIs tend to immediately form precipitants in conventional media, where the reaction becomes inefficient, and tedious purification of the product is necessary. The insolubility typically results from salt formation in PEI. The degree of *N*-alkylation in PEI is also critical to its solubility. If the PEI core is sufficiently alkylated with apolar cetyl chains, the resulting macrosurfactant typically demonstrates good solubility in organic solvents. However, if limited cetyl chains are introduced to one PEI, the resulting product appears to be poorly soluble in either polar or apolar media, possibly because of the strong complementary interactions among the polar cores. Several methods, including well-defined ones [33], have been developed for the synthesis of PEI macrosurfactants [25,26,27,28,29,30,31,32,33,34]. The reaction between epoxy compounds and primary or secondary amino groups is mild, efficient, and does not release side products. When the epoxy-amine reaction is applied to PEI modification, quantitative PEI macrosurfactants with designed *N*-alkylation degrees can be obtained [33], even if the epoxy compound is a macromolecule [33]. PEI macrosurfactants have numerous residual active amines in the core (Figure 1).

## 3. PEI Macrosurfactants as Free Molecular Nanocapsules

**Figure 2:**
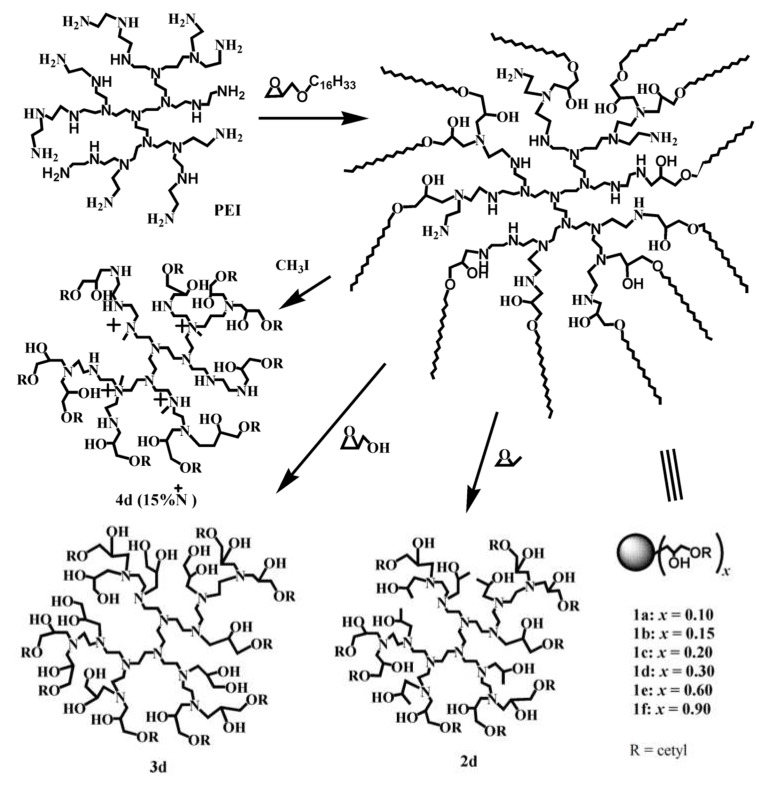
Synthesis of PEI macrosurfactants with different shell densities and core structures [33].

**Figure 3:**
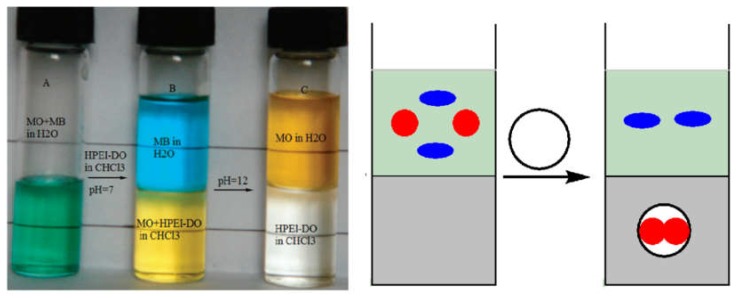
(**Left**) When HPEI-DO (PEI alkylated by dodecyloxirane) in chloroform is shaken with anionic methyl orange (MO) and cationic methyl blue (MB) in water at pH 7, the anionic dye MO is transferred to the bottom chloroform layer, while the cationic MB remains intact in the upper aqueous layer. If the pH is increased to 12, then MO is released into the water layer. (**Right**) Charge-selective encapsulation is responsible for dye separation [32].

PEI can be quantitatively alkylated with glycidyl cetyl (**1a**–**1f**, Figure 2), and up to 90% of the amino protons can be replaced with cetyl groups [33]. A high degree of *N*-alkylation leads to a high possibility of PEI macrosurfactants existing as free molecular nanocapsules [33]. Any of the PEI macrosurfactants in **1a**–**1f** could undergo highly charge-selective guest encapsulation and be used for separating anionic–cationic organic mixtures. The multifunctional core could also be further engineered (**1d**–**4d**, Figure 2) for guest selectivity. Quaternization of the PEI core (**4d**) did not favor charge selectivity and even decreased it, which can be attributed to the van de Waals complement. Evidence showed that the guest selectivity in **1d**–**4d** is related to H-bonding, dipolar–dipolar interactions, and dispersion forces with a guest molecule. For example, **2d** showed higher affinity to methyl orange than **3d**, because methyl orange is both anionic and largely apolar, which is preferred for complementing with less-polar **2d**. Tailoring the core of a molecular nanocapsule can lead to fine differentiation of very similar guests of rose bengal/erythrosine B/eosin Y [35,36]. Deprotonation of PEI leads to the considerable release of encapsulated guests after increasing the pH level [32]. Moreover, molecular nanocapsules are durable and well pH-recyclable, because no acid- or base-labile bonds are present. Chen et al. [30] found that dendritic topology is critical to the guest encapsulation of dendritic molecular nanocapsules, because the linear counterpart cannot efficiently encapsulate similar guests. Alkylated PEI has charge-selective guest encapsulation. Figure 3 shows 100% separated anionic–cationic guests and that the encapsulated dye could be released by pH adjustment, as monitored with a Uv/vis spectrometer [32]. In case the dye is of small size, the encapsulation or releasing equilibration takes seconds to hours; if the dye is of large size, it takes days.

## 4. Direct Dictation of PolyHIPE Surface with PEI Macrosurfactants

**Figure 4:**
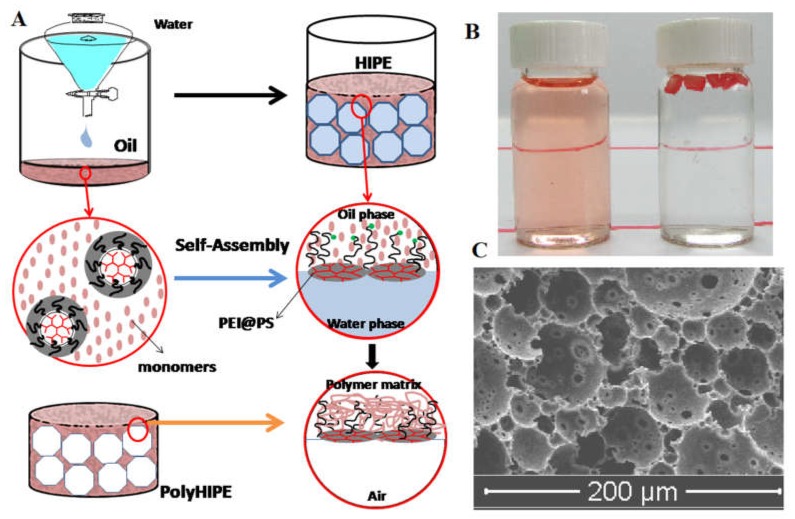
Schematic of dendritic macrosurfactants (PEI@PS)-mediated polyHIPE (**A**), adsorption of Congo Red by the polyHIPE (**B**), and (**C**) SEM micrograph of the polyHIPE [3]. Reproduced with permission from Ye et al., J. Mater. Chem. A; published by the Royal Society of Chemistry, 2015 [3].

Chemical functionalization of a preformed inert polyHIPE is tedious and costly. Physical functionalization has been recently performed using HIPE. Ye et al. [3] directly prepared a functional polyHIPE with a polystyrene (PS)-alkylated PEI (PEI@PS) as macrosurfactant (Figure 4). PEI@PS acted as a HIPE stabilizer and subsequently dictated the surface of the as-prepared polyHIPE. The PS of PEI@PS was responsible for adhering to the polyHIPE matrix, whereas the functional PEI was responsible for guest adsorption. PEI@PS carried no pH-labile bond, indicating that the polyHIPE is chemically durable and has good recyclability properties. Dendritic PEI@PS also showed lower viscosity than its linear counterpart, which is favorable for HIPE [2]. In addition, polyHIPE with a patchy surface was obtained when two kinds of dendritic macrosurfactants were simultaneously charged in a HIPE system [4]. The polyHIPE could simultaneously remove distinctly different guest species from water because of this patchy surface.

**Figure 5:**
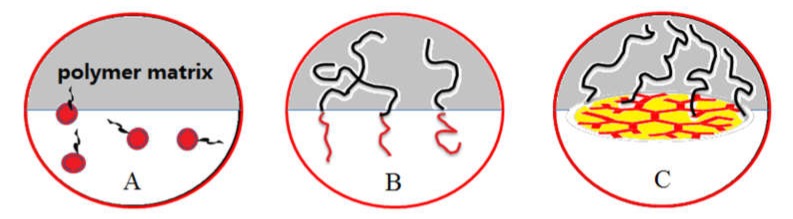
Schematic of small surfactant (**A**), block copolymer (**B**), and (**C**) PEI macrosurfactant-mediated unit of an emulsion.

PEI@PS had a much higher molecular weight than small or diblock copolymer surfactants and did not leave the matrix (Figure 5). For quantitative understanding, one can refer to the dependence of the life span (L) of an assembly on the molecular weight (*M*_n_) of its building blocks through the relationship L = e*^M^*^n^ [46]. Considering that PEI@PS has a *M*_n_ = 10^5^ Dalton, which is much larger than that of a small surfactant (*M*_n_ = 10^2^ Dalton) or a block copolymer (*M*_n_ = 10^4^ Dalton), the stability of a dendritic macrosurfactant can be exponentially improved. This finding was supported by the results of experiments, which showed good recyclability and the absence of any deterioration in the corresponding adsorbents.

## 5. PEI Macrosurfactant-Aided Metal Nanoparticle-Dictated PolyHIPE

**Figure 6:**
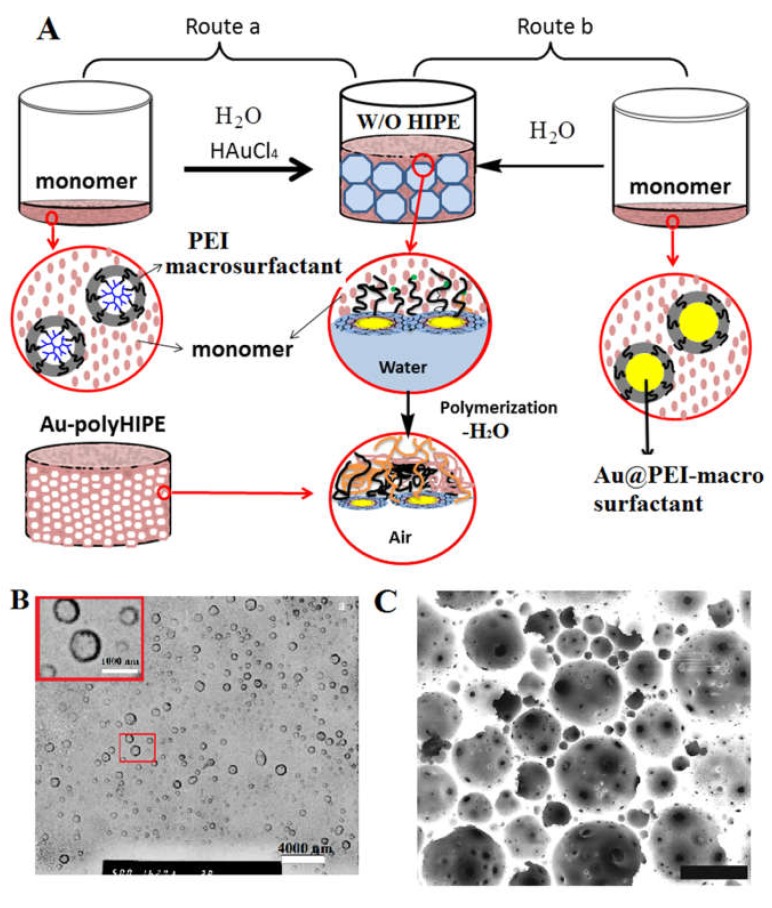
(**A**) Schematic of two routes for PEI macrosurfactant-mediated synthesis of gold nanoparticle-dictated polyHIPE: (a) chloroauric-containing water is dispersed in PEI-macrosurfactant-containing monomer and (b) water is dispersed in Au@PEI-macrosurfactant (gold nanocomposite)-containing monomer; (**B**) TEM micrograph of Au@PEI-macrosurfactant stabilized emulsion; and (**C**) SEM micrograph of polyHIPE with the surface dictated by gold nanoparticles [5]. The scale bar is 4000 nm (1000 nm inset) for (**B**) and 20 μm for (**C**). Reproduced with permission from Ye et al. J. Mater. Chem. A; published by the Royal Society of Chemistry, 2015 [5].

PEI can act as a multiligand of noble metal nanoparticles, which are well-known catalysts. Ye et al. [5] adopted the PEI@PS-mediated HIPE process to prepare gold nanoparticle-dictated polyHIPE by charging gold ion species during the water phase. Upon polyHIPE formation, the gold ions were simultaneously reduced to gold nanoparticles, with PEI acting as the ligand and reducer (Figure 6). Alternatively, PEI@PS was loaded with gold nanoparticles in advance, and the resulting product was used as HIPE surfactant to similarly prepare polyHIPE. In both cases, open-cellular polyHIPEs were obtained, and both showed good catalytic and recyclability properties. Liu et al. [6] found that if the polyHIPE matrix was made of flexible polymers rather than brittle PS, no chalky fragment would form during catalytic application, which favors the recovery of platinum nanoparticle-dictated catalytic materials. Wan et al. [7] showed that when trace and optimized amounts of thiol groups are covalently introduced onto PEI, highly durable platinum nanoparticles can be obtained, and the catalytic activity is only slightly reduced by the thiol groups.

The open-cellular structure of a polyHIPE is critical to the accessibility of the surface and the catalytic sites. PolyHIPEs mediated with small molecular surfactant generally yield an open-cellular macroporous polymer, but the mechanism remains unclear. Block copolymer and dendritic macrosurfactants also typically lead to open-cellular polyHIPEs. One study attributed the mechanism to volume contraction upon gel formation [47]. Other studies suggested the role of phase separation and vacuum induction [48]. Li et al. [49] suggested that pore formation is not related to vacuum and attributed it to the combination of volume contraction and surfactant migration upon gelation. Closed cellular polyHIPE is typically obtained when nanoparticles are used as the stabilizer of a HIPE system (called Pickering emulsion). Ye et al. [5] and Liu et al. [6] obtained gold (or platinum) nanoparticle-mediated polyHIPEs with an open-cellular structure (which is critical to accessing catalytic sites). The free PEI macrosurfactants in the system possibly played a critical role, because when minor small surfactants are present in a Pickering emulsion system, open-cellular rather than closed-cellular polyHIPE is obtained [50,51,52,53,54,55].

## 6. PEI Macrosurfactant-Aided AminopolycarboxylicAcid-Dictated PolyHIPE for Metal Ion Adsorption

**Figure 7:**
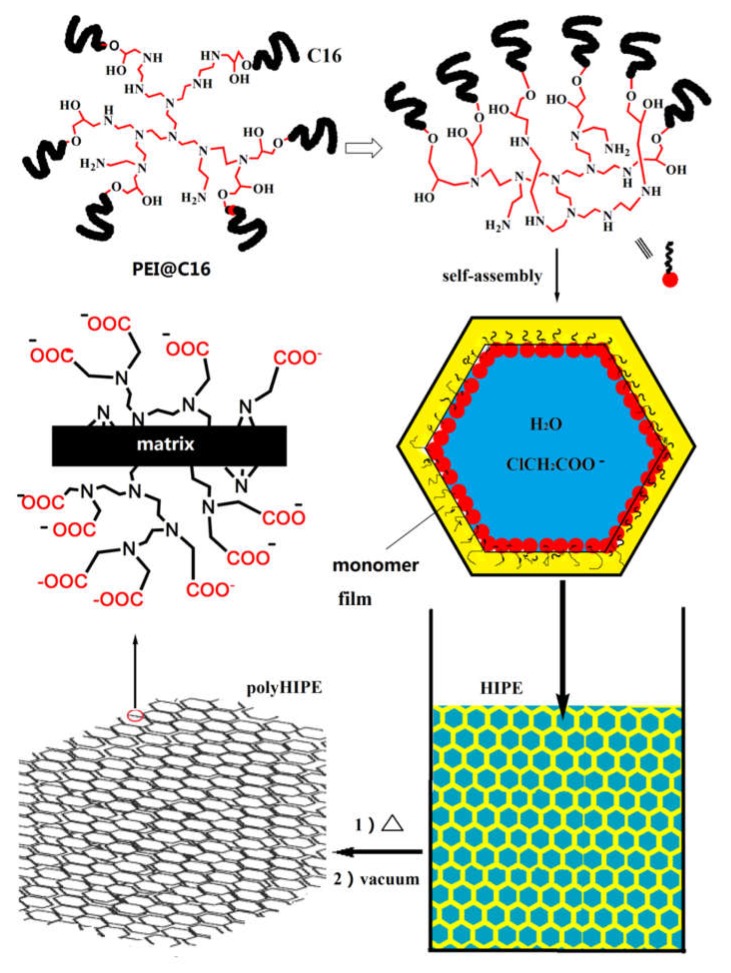
Schematic of PEI-macrosurfactant-mediated synthesis of aminopolycarboxylic acid-dictated polyHIPE. Reproduced with permission from Weng et al. Mater. Chem. Frontiers; published by the Royal Society of Chemistry, 2020 [57].

Aminopolycarboxy acids (APAs) are known for their extremely high affinity for a wide spectrum of toxic and cationic metal species. Ethylenediaminetetraacetic acid and diethylenetriaminepentaacetic acid are typical members of the APA family. Many researchers have tried to attach APAs on a supported porous matrix [56], mainly via solid-phase reactions. After dictation on the polyHIPE surface, the active amino groups of PEI macrosurfactants represent a useful engineering platform. Weng et al. [57] very recently developed a physically aided and one-pot HIPE process for APA-dictated polyHIPE (Figure 7). The self-assembly of PEI macrosurfactant stabilized the HIPE and rendered the active amino groups arrayed along the oil/water interface, thereby leading to their transformation into APAs. The resulting polyHIPE can eliminate various heavy metal species at trace amounts in water. This adsorbent has the advantages of cost-effective preparation, high durability, and good metal-eliminating ability. However, the linear counterpart of dendritic PEI macrosurfactant cannot be applied to the same process due to its very poor solubility, which stems from intermolecular complementary interaction-induced aggregation. In the case of dendritic macrosurfactants, the intramolecular complement is predominant because of its globular molecular topology.

## 7. Conclusions and Outlook

Dendritic PEI macrosurfactants could dictate the surface of an in-situ-prepared open-cellular polyHIPE, a typical kind of macroporous polymer, by using a HIPE templating technique. The dendritic macrosurfactants showed lower viscosity and better solubility than their linear counterparts, indicating their enhanced performance in a HIPE process. They robustly attached to the polyHIPE matrix regardless of the physical adhesion nature. PolyHIPEs with surfaces dictated by functional PEI macrosurfactants could act as a well-recyclable adsorbent. Catalytic metal nanoparticles could be facilely introduced onto the surface of a polyHIPE through the help of PEI macrosurfactants, thereby making these nanoparticles readily recyclable catalysts. With the residual active amino groups on the polyHIPE surface, the polyHIPE was transformed into aminopolycarboxylic acid, capable of eliminating various heavy metal species from water.

The application of most polyHIPEs is related to their open-cellular structure and surface functions when adsorption and catalysis are involved. In this respect, dendritic macrosurfactants generally lead to an open-cellular structure and a designable functional surface. Moreover, dendritic macrosurfactants demonstrate a good open-cellular structure after being partly loaded with metal nanoparticles. Most functional inorganic–organic nanocomposite particles are amphiphilic; they possibly could be similarly immobilized on a polyHIPE surface.

## References

[1] Zhang S.M., Wang D.G., Pan Q.H., Gui Q.Y., Liao S.L., Wang Y.P. (2017). Light-Triggered CO_2_ Breathing Foam via Nonsurfactant High Internal Phase Emulsion. ACS Appl. Mater. Interfaces.

[2] Feng Y.Y., Zhang X.X., Jin M., Wan D.C. (2017). Dendritic Macrosurfactant-Decorated PolyHIPE as a Highly Efficient and Well Recyclable Scavenger of Micropollutants in Water: Topological Effect. J. Polym. Sci. Part A Polym. Chem..

[3] Ye Y.L., Wan D.C., Du J., Jin M., Pu H.T. (2015). Dendritic Macrosurfactant Mediated Porous Monolith for Eliminating Organic Micropollutants from Water. J. Mater. Chem. A.

[4] Feng Y.Y., Wan Y.J., Jin M., Wan D.C. (2018). Large-scale preparation of 3D patchy surface with dissimilar dendritic macrosurfactants. Soft Matter.

[5] Ye Y.L., Jin M., Wan D.C. (2015). One-Pot to Porous Monolith-Supported Gold Nanoparticles as a Well Recyclable Catalyst. J. Mater. Chem. A.

[6] Liu H.H., Wan D.C., Du J., Jin M. (2015). Dendritic Macrosurfactant Mediated One-Pot Preparation of 3D Pt Nanoparticles-Decorated PolyHIPE as a Durable and well Recyclable Catalyst. ACS Appl. Mater. Interfaces.

[7] Pulko I., Wall J., Krajnc P., Cameron N.R. (2010). Ultra-High Surface Area Functional Porous Polymers by Emulsion Templating and Hypercrosslinking: Efficient Nucleophilic Catalyst Supports. Chem.-Eur. J..

[8] Wan Y.J., Feng Y.Y., Wan D.C., Jin M. (2016). Polyamino Macrosurfactant Mediated Supporting of Platinum Nanoparticles on PolyHIPE as an over 1500-Time Recyclable Catalyst. RSC Adv..

[9] Viswanathan P., Ondeck M.G., Chirasatitsin S., Ngamkham K., Reilly G.C., Engler A.J., Battaglia G. (2015). 3D surface topology guides stem cell adhesion and differentiation. Biomaterials.

[10] Taylor-Pashow K.M.L., Pribyl J.G. (2019). PolyHIPEs for Separations and Chemical Transformations: A Review. Sol. Extract. Ion Exch..

[11] Bartl H., Bonin W.V. (1962). Uber die Polymerisation in Umgekehrter Emulsion. Makromol. Chem..

[12] Pulko I., Krajnc P. (2012). High Internal Phase Emulsion Templating—A Path to Hierarchically Porous Functional Polymers. Macromol. Rapid Commun..

[13] Zhang T., Sanguramath R.A., Israel S., Silverstein M.S. (2019). Emulsion Templating: Porous Polymers and Beyond. Macromolecules.

[14] Silverstein M.S. (2014). Emulsion-templated porous polymers: A retrospective perspective. Polymer.

[15] Wu D.C., Xu F., Sun B., Fu R.W., He H.K., Matyjaszewski K. (2012). Design and Preparation of Porous Polymers. Chem. Rev..

[16] Brun N., Ungureanu S., Deleuze H., Backov R. (2011). Hybrid Foams, Colloids and beyond: From Design to Applications. Chem. Soc. Rev..

[17] Kimmins S.D., Cameron N.R. (2011). Functional Porous Polymers by Emulsion Templating: Recent Advances. Adv. Funct. Mater..

[18] Kovacic S., Preishuber-Pflugl F., Pahovnik D., Zagar E., Slugovc C. (2015). Covalent incorporation of the surfactant into high internal phase emulsion templated polymeric foams. Chem. Commun..

[19] Zhang T., Silverstein M.S. (2018). Microphase-Separated Macroporous Polymers from an Emulsion-Templated Reactive Triblock Copolymer. Macromolecules.

[20] Bancroft W.D. (1913). The theory of emulsification V. J. Phys. Chem..

[21] van Hest J.C.M., Delnoye D.A.P., Baars M.W.P.L., van Genderen M.H.P., Meijer E.W. (1995). Polystyrene-dendrimer amphiphilic block-copolymers with a generation-dependent aggregation. Science.

[22] Zhou Y.F., Yan D.Y. (2004). Supramolecular self-assembly of giant polymer vesicles with controlled sizes. Angew. Chem. Int. Ed..

[23] Rosen B.M., Wilson C.J., Wilson D.A., Peterca M., Imam M.R., Percec V. (2009). Dendron-Mediated Self-Assembly, Disassembly, and Self-Organization of Complex Systems. Chem. Rev..

[24] Jiang W.F., Zhou Y.F., Yan D.Y. (2015). Hyperbranched polymer vesicles: From self-assembly, characterization, mechanisms, and properties to applications. Chem. Soc. Rev..

[25] Kramer M., Stumbe J.F., Turk H., Krause S., Komp A., Delineau L., Prokhorova S., Kautz H., Haag R. (2002). pH-responsive molecular nanocarriers based on dendritic core-shell architectures. Angew. Chem. Int. Ed..

[26] Chen Y., Shen Z., Pastor-Perez L., Frey H., Stiriba S.E. (2005). Role of topology and amphiphilicity for guest encapsulation in functionalized hyperbranched poly(ethylenimine)s. Macromolecules.

[27] Wan D.C., Lai Y., Jin M., Pu H.T. (2011). Selective Encapsulation of Ionic Dyes by Core/Shell Amphiphilic Macromolecules Derived from Hyperbranched Polyethylenimine:Properties through Structures. Macromol. Chem. Phys..

[28] Wan D.C., Jin M., Pu H.T. (2014). Charge-Selective Separation and Recovery of Organic Ions by Polymeric Micelles. J. Polym. Sci. Part B Polym Phys..

[29] Antonietti L., Aymonier C., Schlotterbeck U., Garamus V.M., Maksimova T., Richtering W., Mecking S. (2005). Core-shell-structured highly branched poly(ethylenimine amide)s: Synthesis and structure. Macromolecules.

[30] Liu H.J., Chen Y., Zhu D.D., Shen Z., Stiriba S.E. (2007). Hyperbranched polyethylenimines as versatile precursors for the preparation of different type of unimolecular micelles. React. Funct. Polym..

[31] Cao X.L., Li Z.Q., Song X.W., Cui X.H., Cao P.F., Liu H.J., Cheng F., Chen Y. (2008). Core-shell type multiarm star poly(epsilon-caprolactone) with high molecular weight hyperbranched polyethylenimine as core: Synthesis, characterization and encapsulation properties. Eur. Polym. J..

[32] Wan D.C., Pu H.T., Cai X.Y. (2008). Separation Promoted by Molecular Recognition of a Core Engineered Macromolecular Nanocapsule. Macromolecules.

[33] Wan D.C., Wang G.C., Pu H.T., Jin M. (2009). Can Nonspecific Host-Guest Interaction Lead to Highly Specific Encaspulation by a Supramolecular Nanocapsule?. Macromolecules.

[34] Wan D.C., Yuan J.J., Pu H.T. (2009). Macromolecular Nancapsule Derived from Hyperbranched Polyethylenimine (HPEI): Mechanism of Guest Encapsulation versus Molecular Parameters. Macromolecules.

[35] Wan D.C., Pu H.T., Jin M. (2010). Highly Specific Molecular Recognition by a Roughly-Defined Supramolecular Nanocapsule: A Fuzzy Recognition Mechanism. Macromolecules.

[36] Wan D.C., Pu H.T., Jin M., Wang G.W., Huang J.L. (2011). Supramolecular Fuzzy Recognition Leads to Effective Differentiation of Similar Molecules. J. Polym. Sci. Part A Polym. Chem..

[37] Wan D.C., Jin M., Pu H.T., Pan H.Y., Chang Z.H. (2010). Enhancing the Unimolecularity and Control for Guest Release of a Macromolecular Nanocapsule via Core Engineering. React. Funct. Polym..

[38] Viswanathan P., Johnson D.W., Hurley C., Cameron N.R., Battaglia G. (2014). 3D Surface Functionalization of Emulsion-Templated Polymeric Foams. Macromolecules.

[39] Gui H.G., Guan G.W., Zhang T., Guo Q.P. (2019). Microphase-separated, hierarchical macroporous polyurethane from a nonaqueous emulsion-templated reactive block copolymer. Chem. Eng. J..

[40] Zhang T., Silverstein M.S. (2019). Robust, highly porous hydrogels templated within emulsions stabilized using a reactive, crosslinking triblock copolymer. Polymer.

[41] Mathieu K., Jerome C., Debuigne A. (2015). Influence of the Macromolecular Surfactant Features and Reactivity on Morphology and Surface Properties of Emulsion-Templated Porous Polymers. Macromolecules.

[42] Li W.W., Yu Y., Lamson M., Silverstein M.S., Tilton R.D., Matyjaszewski K. (2012). PEO-Based Star Copolymers as Stabilizers for Water-in-Oil or Oil-in-Water Emulsions. Macromolecules.

[43] Xie G.J., Krys P., Tilton R.D., Matyjaszewski K. (2017). Heterografted Molecular Brushes as Stabilizers for Water-in-Oil Emulsions. Macromolecules.

[44] Xu Z.S., Ford W.T. (2002). Polystyrene latexes containing poly(propyleneimine) dendrimers. Macromolecules.

[45] Yi C.F., Shen Y.H., Deng Z.W., Xu Z.S., Ford W.T. (2004). Emulsion polymerization of styrene containing dendrimer PAMAM. Acta Polym. Sin..

[46] Christian D.A., Tian A.W., Ellenbroek W.G., Levental I., Rajagopal K., Janmey P.A., Liu A.J., Baumgart T., Discher D.E. (2009). Spotted vesicles, striped micelles and Janus assemblies induced by ligand binding. Nat. Mater..

[47] Cameron N.R., Sherrington D.C., Albiston L., Gregory D.P. (1996). Study of the formation of the open cellular morphology of poly(styrene/divinylbenzene) polyHIPE materials by cryo-SEM. Colloid Polym. Sci..

[48] Menner A., Bismarck A. (2006). New evidence for the mechanism of the pore formation in polymerising High Internal Phase Emulsions or why polyHIPEs have an interconnected pore network structure. Macromol. Symp..

[49] Li C.H., Jin M., Wan D.C. (2019). Evolution of a Radical-Triggered Polymerizing High Internal Phase Emulsion into an Open-Cellular Monolith. Macromol. Chem. Phys..

[50] Ikem V.O., Menner A., Horozov T.S., Bismarck A. (2010). Highly Permeable Macroporous Polymers Synthesized from Pickering Medium and High Internal Phase Emulsion Templates. Adv. Mater..

[51] Zhu W.X., Zhu Y., Zhou C., Zhang S.M. (2019). Pickering emulsion-templated polymers: Insights into the relationship between surfactant and interconnecting pores. RSC Adv..

[52] Tebboth M., Kogelbauer A., Bismarck A. (2015). Liquid-Liquid Extraction within Emulsion Templated Macroporous Polymers. Ind. Eng. Chem. Res..

[53] Kovacic S., Anzlovar A., Erjavec B., Kapun G., Matsko N.B., Zigon M., Zagar E., Pintar A., Slugovc C. (2014). Macroporous ZnO Foams by High Internal Phase Emulsion Technique: Synthesis and Catalytic Activity. ACS Appl. Mater. Interfaces.

[54] Hua Y., Zhang S.M., Zhu Y., Chu Y.Q., Chen J.D. (2013). Hydrophilic polymer foams with well-defined open-cell structure prepared from Pickering high internal phase emulsions. J. Polym. Sci. Part A Polym. Chem..

[55] Hua Y., Zhang S.M., Chen J.D., Zhu Y. (2013). Switchable release and recovery of nanoparticles via a Pickering-emulsion-templated porous carrier. J. Mater. Chem. A.

[56] Repo E., Warch J.K., Bhatnagar A., Mudhoo A., Sillanpaa M. (2013). Aminopolycarboxylic Acid Functionalized Adsorbents for Heavy Metals Removal from Water. Water Res..

[57] Weng S.Q., Jin M., Wan D.C. (2020). Macrosurfactant Mediated, Aminopolycarboxy Acid Dictated and Open-cellular Adsorbent for Removing Metal Micropollutants from Water. Mater. Chem. Front..

